# Re-taking the field: resuming in-person fieldwork amid the COVID-19 pandemic

**DOI:** 10.1515/lingvan-2021-0155

**Published:** 2024-09-13

**Authors:** Alice Idone, Lisa Gasner, Giulia Donzelli, Adriano Salvi, Michele Loporcaro

**Affiliations:** Institute of Romance Studies, 27217 University of Zurich, Zurich, Switzerland

**Keywords:** fieldwork, COVID-19 pandemic, Italy, Lombard dialects

## Abstract

Italy was among the first countries in Europe to be heavily impacted by the outbreak of COVID-19. Northern Italy and Lombardy, in particular, were the most affected areas. In September 2021, the team of “AIS, the digital turn” (AISdt), a project whose objectives include collecting data in 50 locations in Italy, across Lombardy and Piedmont, resumed in-person data collection. Given the field of the investigation and the profile required for the informants (speakers over 60), the decision came only after a long examination of the right conditions and the necessary measures. The paper is intended as a reference text of field investigations amid the pandemic: based on the experience of the AISdt project, it recounts the preparation required by the new modality of fieldwork, the protocols put in place to ensure the safety of all participants before and during the interviews, and the response received from the informants.

## 1 Introduction

Italy was among the first countries in Europe impacted by the outbreak of COVID-19, and one of the most affected (Wikipedia 2021). From January 2020 – when the presence of the virus was first confirmed – up to November 2021, the total number of cases reported by the authorities exceeded 5 million (Protezione Civile 2021a). Northern Italy, and Lombardy in particular, were the earliest and most strongly affected parts of the country. According to the Italian Ministry of Health, nearly 20 % of all cases reported in Italy (930,321 as at 29 November 2021) were recorded in Lombardy (Statista 2021). The datum is over-proportional, given that the region accounts for 16.7 % of the overall population in Italy (10,060,574 inhabitants out of 60,317,000), as measured by the Italian institute for statistics (ISTAT 2020), on 1 January 2020, before the outbreak of the pandemic.

As a reaction to the surge of cases, the Italian government approved strict containment measures from March 2020.1A comprehensive report of all measures issued by the Italian Council of Ministers from the beginning of the pandemic is available at Presidenza del Consiglio dei Ministri (2021). Only in August 2021, in the wake of a widespread vaccination campaign, did the Government ease the lockdown. As of November 2021, 84.5 % of the Italian population was fully vaccinated (Ministero della Salute 2021), and the infection rate was lower than the European average (Protezione Civile 2021b; Worldometer 2021). Restrictions were applied at regional and city levels, based on the local epidemiological situation, and economic and social activities were subject to the possession of an EU Digital COVID Certificate (EUDCC), also known as a “green pass”. Hence, in order to access workplaces, education and health facilities, and recreational venues, as well as to use most means of transportation and attend many public and private indoor and outdoor events, citizens over 12 were asked to hold proof of having been fully vaccinated against COVID-19, of having recovered from the disease, or of having received a negative test result within the previous 48 h. The use of a face mask was mandatory in indoor public places, whereas it was no longer mandatory outdoors in most cities – although in Lombardy, mayors and prefects reintroduced its use in the largest cities at the end of November 2021 – but people were asked to always carry a mask with them and wear it whenever the interpersonal safety distance of one metre could not be guaranteed. Face masks were also strongly recommended in private homes, when in the presence of non-cohabiting people.

In October 2019, when the funding request for the project “AIS, the digital turn” (AISdt; Loporcaro et al. 2021a) was submitted, no one could have foreseen the impending pandemic; nor could anyone have anticipated that setting as a goal the collection of data in 50 locations across Lombardy and Piedmont would have complicated matters further. Having postponed the starting date of the project to April 2021, the team of AISdt started its fieldwork campaign in September 2021. The decision came after a long period of evaluation of the risks. It was encouraged by the increasing rate of vaccination and the ongoing re-opening of areas of public life by the Italian government. At the same time, this choice required a strong commitment from the research team. In the months prior to the start of the data collection, the members of the AISdt project gathered the necessary information and consulted with institutions and experts in order to develop a data collection protocol that would guarantee the safety of informants and researchers.

**Figure 1:**
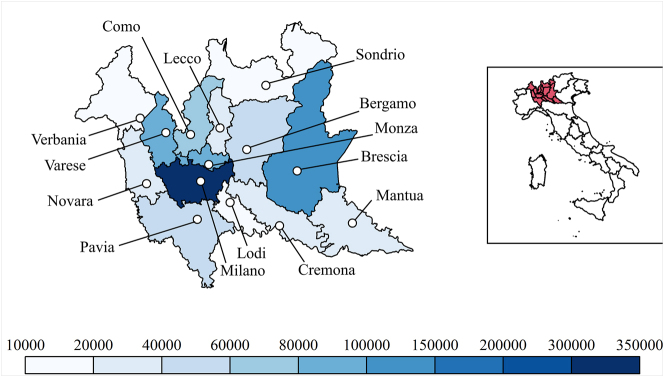
Total number of COVID-19 cases recorded in the area of the AISdt fieldwork in the period from February 2020 to November 2021. COVID-19 data from Protezione Civile (2021c); map data from https://gadm.org/data.html.

The paper shares the solutions conceived to resume in-person collection for the project, at the end of the second year of the pandemic, and in a region hit brutally by COVID-19 (see Figure 1). The experience and the information on regulations refer to the period between September and November 2021. It is meant as a sort of logbook of the first investigations in the pandemic setting. It recounts the preparation behind the new modality of fieldwork (Section 3), the protocols put in place to ensure the safety of all participants before and during the interviews (Section 4), and the response of informants and interviewers (Section 5). Well aware that the collection set-up heavily relates to the ultimate aim of the investigation, we will first present the research goal of the AISdt project (Section 2). However, each section will emphasize the most replicable aspects of our fieldwork, aiming at providing general insights into data collection in the context of pandemic events.

## 2 The fieldwork for the project “AIS, the digital turn”

AISdt is an ongoing research project funded by the Swiss National Science Foundation (SNSF, grant 100012_192259, period 2021–2025), and carried out at the Institute of Romance Studies of the University of Zurich. Its area of research is the documentation of non-standard Italo- and Raeto-Romance varieties, with a special focus on the dialects of the Lombard group.2We use the term (local) “dialect” and “non-standard variety” interchangeably following the consolidated tradition in Italian studies. For those not familiar with the Italian linguistic landscape, note that the dialects spoken in Italy are not dialects of Italian, but non-standardized languages independently evolved from Latin and the local substratum languages spoken in the territory. For more information on the linguistic classification of Italian varieties, see Pellegrini (1977), Maiden and Parry (1997), and Loporcaro (2013), among others.

AIS is the acronym for *Atlante linguistico ed etnografico dell’Italia e della Svizzera meridionale* (Jaberg and Jud 1928–1940), that is, the linguistic and ethnographic atlas of Italy and southern Switzerland. That monumental work, conceived by the Swiss linguists Karl Jaberg and Jakob Jud and published in eight volumes, presents the data collected at 407 locations, providing a detailed survey of their material culture and language between 1919 and 1930. As such, the AIS is still an essential reference for Italo- and Raeto-Romance studies.

**Figure 2:**
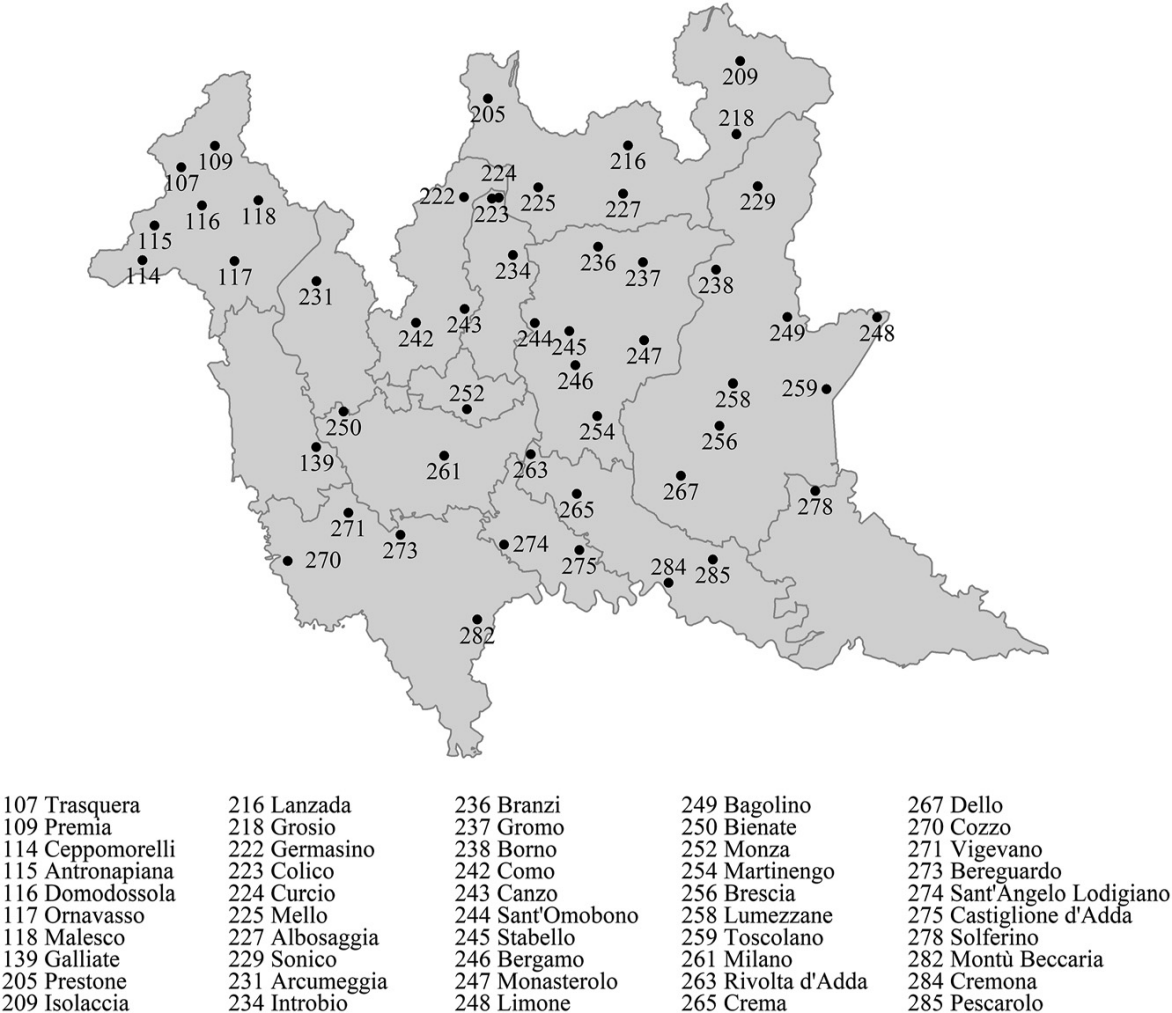
Geographic location of the data collection sites of the AISdt project. Map data from https://gadm.org/index.html.

The project AISdt is working to ensure that the AIS renews its status as a major research tool by updating it in terms of the accessibility and topicality of the data. As the research team of the AISdt, we are working to provide a fully digital version of the AIS, making all data available and queryable online, and freely downloadable as a database; hence the mention of “the digital turn” in the title.3The digitalization of the original atlas is being achieved by means of optical character recognition software custom-developed by Graziano Tisato (CRN, Padova). In compliance with the FAIR principles (Wilkinson et al. 2016), the output of the AISdt project is being uploaded as an online database at https://www.ais-reloaded.uzh.ch. In doing so, AISdt is completing the process started by the project “AIS, reloaded” (AISr; Loporcaro et al. 2019, 2021b). Moreover – and more relevantly for the ends of this paper – AISdt is supplementing the content of the original atlas with new data collected in the field. The fieldwork campaign covers 50 AIS data points in Italy where a Lombard dialect is spoken: 42 localities in Lombardy and 8 in eastern Piedmont (see Figure 2).

The goal is to collect a corpus of present-day data directly comparable to that collected for the AIS by Paul Scheuermeier between 1919 and 1925. Present and past data will be available on the same web platform, designed to allow the user to readily observe any linguistic change that may have occurred a century later. Full comparability is achieved by administering the original AIS questionnaire at the same AIS data points, and by keeping the same sample target, that is, documenting the idiolect of one informant at each point. What changes are, of course, the tools and the procedure of the recording.

The new corpus is elicited by means of the free software SpeechRecorder (version 6.5.0; Draxler and Jänsch 2004). Each entry of the questionnaire (1,737 in total) is displayed on a white screen in the Italian translation and/or as a picture. The informant is asked to name the object or a certain concept, or translate a full sentence into their own dialect. Each answer is audio-recorded directly into a laptop connected to an audio interface, which is in turn linked to an external microphone. Additionally, each interview session is video-recorded in order to keep track of any additional comment made by the informant and all the spontaneous interactions with interviewers and third parties attending the interview.

The original AIS questionnaire mirrors the society of the 1920s, especially given the ethnographic vocation of the atlas. Therefore, many of the entries pertain to objects and activities that have long become either obsolete or marginal in today’s society. The lexicon of plantation and breeding farms, forestry, craftmanship, and all related tools is highly represented. Hence, in order to document phenomena that go beyond lexical decay – which is the default scenario in the Italian sociolinguistic set-up – and not to make the interview frustrating for the informant, for the collection of present-day data we recruit speakers over 60 years old with an active dialect competence, preferably with a rural background.4As inferable from Figure 2, the list of locations to be investigated also includes large cities like Milano or Brescia for which the AIS questionnaire is not linguistically ecological. This the case for the AISdt surveys, but was equally true at the time of the investigations for the original AIS. For this reason, very often the tables referring to rural objects and activities do not record any answers for big cities, in the old as well as in the newly collected database. In highly urbanized environments, age and active linguistic competence are considered sufficient criteria for the selection of the informant.

The profile of the informants is one of the key factors that led us to dismiss the option of remote data collection from the outset. Not only could we not assume that they would be sufficiently familiar with computer technology, but we believe that the size of the questionnaire forbids remote or online collection. It requires an average of 15 hours to administer the entire questionnaire. For the AISdt project, data collection by any means other than an in-person modality would have had a major impact on the whole concept of the research and a very high margin of risk and failure. On the other hand, given the average age of our informants, taking to the field amid the COVID-19 pandemic had serious implications as well, as it meant interacting with one of the high-risk groups. For this reason, the decision to resume the fieldwork activities came after a long period of evaluation and constant monitoring of the existing regulations, the infection curve, and the progress of the vaccination campaign. It also came together with the elaboration of a strict protocol put in place to ensure the maximum safety of all participants before and during the interviews.

## 3 Fieldwork amid the COVID-19 pandemic: preparatory work

### 3.1 Getting informed about the regulations

When planning fieldwork during a pandemic, the first step is to read up on entry requirements and measures in place in the country and region where the investigation will take place. This should be done consistently, as new rules and guidelines can be enforced on very short notice.5In Italy alone up to 26 November 2021, a total of 799 acts were issued to counter the advance of coronavirus: 466 in 2020 and 333 in 2021; an average of about 35.7 per month (Openpolis 2021).

Given the aims of the AISdt project, we have direct experience of the Italian regulations.6Since our fieldwork required travelling between Switzerland and Italy, we found the EU official website and mobile app Re-open EU (https://reopen.europa.eu/en/) particularly useful, as it provided regularly updated information for all EU/EEA countries. Italy adopted very strict measures from the earliest stages of the pandemic and for very long periods (as briefly outlined in Section 1). Up to the end of November 2021, economic and social activities were subject to the possession of the EUDCC. The use of face masks was mandatory in indoor public places, whereas it was no longer mandatory outdoors in most cities, but not everywhere in Italy. Moreover, face masks were required whenever the interpersonal safety distance of one metre could not be guaranteed, and they were strongly recommended also in private residences in presence of people from different households.

As we have come to know, restrictions on public and private gatherings and movement are in principle adjusted based on different factors, depending on the infection spread curve. While there is now broad consensus in the scientific community as to what protocols are effective in limiting the spread of coronaviruses, governments are free to make individual choices as to the severity of the measures to be enforced.7For an overview of the severity of the measures active worldwide, one may refer to the WHO portal (WHO 2021b), which, however, is not intended to be exhaustive. In countries where some measures are not taken or are eased, for example with respect to the use of face masks, linguists in the field may be faced with the choice of whether or not to adopt stricter additional measures to protect themselves and informants.

### 3.2 Finding the right location and selecting the right equipment

In times of pandemic, data collection methods and tools may need to be reconsidered. When choosing a location for interviews, the researcher should opt for an experimental setting that allows everyone to respect social distancing requirements. If the purpose of the investigation and the season allow, outdoor locations should be preferred; obviously, no phonetician would ever go for this solution due to ambient noise. Before COVID-19, at least in Italy and especially in small villages, interviews at the home of the informant were a common practice, but under the circumstances we decided to avoid that for several reasons. The pandemic strongly influenced general perceptions of space and safety, and during lockdowns home became the safe place for people, and we needed to respect that. Moreover, from a methodological perspective, working as a guest in the home of an informant may affect the control that the linguist has on preventive measures (e.g., disinfection of room surfaces, ventilation of the environment, interactions with additional people, and so on). Based on our experience, it is recommended to ask a local contact person or institution (e.g., municipal government, library, local association, parish church, etc.) to help identify a suitable location, like a spare room that is not liable to be accessed by any other persons during the whole duration of the fieldwork.

The choice of the right equipment should not be underestimated either, as any object used during interviews should be easily disinfected. This concerns, in particular, the microphone and every device that the informant and/or the interviewer will touch. In many work environments, the problem of saliva droplets on microphones has been addressed by using disposable non-permeable plastic covers. This solution could be applied to linguistic fieldwork too, for those studies where a high level of sound quality is not essential. However, a more efficient solution is to opt for microphones that can be disinfected easily. Thus, the disinfecting of a head-basket microphone is more demanding if there is a foam shield between the capsule and the metal grid; a mic windscreen should be avoided unless you are absolutely certain to be able to properly wash the furry or foamy component after each interview. With the surge of the virus, many manufacturers of recording equipment supported customers with hygiene guides that account for specific models (see, for example, Sennheiser 2021).8For the equipment used for the AISdt project we gratefully acknowledge the assistance of the staff of Phonogram Archives of the University of Zurich, in the person of Camilla Bernardasci, in the selection and maintenance of devices suitable for fieldwork in these unprecedented times. Proper instructions should be sought for headphones as well, especially for foam ear pads. As for the sanitation of notebooks, cameras, and other electronic external devices, we noticed that the easiest way to keep them clean is to wipe them regularly with disinfecting wipes. If the surface is particularly delicate, as in the case of monitors, tablets, and lenses, an alternative is to wrap them with a cleanable or disposable film.

The most distinctive units of fieldwork equipment during a pandemic are items used for disinfecting, and personal protective equipment (PPE). These should include at least protective masks, disinfectant gel, and disinfectant wipes. Depending on the arrangement of participants and the collection methods, the linguist might consider using gloves, goggles, and face shields. As it will be described more in detail in Section 4, for the fieldwork of the AISdt project, the hygiene measures consisted of FFP2 masks, disinfectant gel and wipes, face shields, and SARS-CoV-2 antigen self-tests.

As a general insight, it should be mentioned that pursuing this modality of fieldwork has an impact on the budget: new proposals should consider it, and projects funded before the start of the pandemic must cope and be sure to allocate the right resources to it. The costs not only relate to the purchase of disinfectant and PPE equipment, but also to the availability of funds to test the researchers (and, ideally, the informants as well) on a regular basis, or, as the case may be, to be able to pay for longer stays to comply with self-isolation requirements.

### 3.3 Updating the informed consent form

A further important aspect of pandemic fieldwork concerns the responsibilities of interviewers and interviewees participating in it. The informed consent form must be updated in order to acknowledge the sanitary emergency. In addition to the sections relating to purpose of the research, procedure, and data treatment, the information sheet should include a paragraph describing the risks connected to the transmission of – in this case – the new SARS-CoV-2, and the preventive measures put in place by the research team to ensure the safety of all parties involved. The risk assessment depends upon the nature and type of data collection, and precautionary measures should be tailored to the specific collection set.

Moreover, the consent certificate must include a declaration signed by the informant, with explicit reference to the fact that they do not have any COVID-19 symptoms and have not engaged in any behaviour that could lead to the transmission of the virus.9This clause can be interpreted as the exclusion of all non-vaccinated persons from the interview. Although there was no mandatory vaccination requirement in any European country at the time, it is the right of the researcher to decide to work only with vaccinated informants. On the basis of the information made available by the authorities, we trusted that the protocols described in Section 3.2 were sufficient to prevent the transmission of COVID-19. As an additional precaution, if faced with the need to include non-vaccinated individuals, we considered asking the informant to show us a negative test result. However, in Italy, from January to March 2022, getting tested soon became insufficient to allow non-vaccinated people to participate in social activities in risk areas, once the “super green pass” was introduced (Winfield 2022).

Here we provide, in English translation, an excerpt from the informed consent form used for the fieldwork of the AISdt project:

[…] The data collection is carried out in compliance with the measures issued by the President of the Italian Republic (Decree-Law no. 175 of 23 July 2021) for safe social activities during the COVID-19 emergency. The people responsible for data collection have all completed the vaccination cycle and will wear FFP2 protective face masks for the entire duration of the interview. The informant cannot wear a face mask for technical reasons but is offered the option of wearing a protective face shield. All equipment is disinfected at the beginning and end of each recording session. […]

In relation to the risks posed by the current health emergency, I declare that:

– I have not experienced symptoms such as fever, fatigue, difficulty in breathing, or dry cough, or other symptoms related to COVID-19 infection in the past 14 days.
– I have not been diagnosed with COVID-19 infection in the last 30 days.
– I have not been in contact with a person diagnosed with COVID-19 infection in the last 30 days.
– I am fully and personally responsible for my own safety and actions while participating in the interview, and I am aware of the modalities of transmission of COVID-19 infection.
– I acknowledge that the research team has put in place all necessary preventive measures aimed to avoid the spread of COVID-19. However, given the nature of the virus, I understand there is an inherent risk of becoming infected with COVID-19.

When writing the consent form, it is of utmost importance to refer to the regulations concerning civil and penal liability in force in the country where the fieldwork is carried out. We found it useful to consult with our research institution’s legal department and with a lawyer in the country where the fieldwork was to take place.

## 4 Logbook of fieldwork amid the COVID-19 pandemic

For the fieldwork of the AISdt project, the preparatory work described in Section 3 was usually carried out at least two weeks in advance of the dates when we intended to hold the interviews. During this time, we focused on finding a contact in the village or city of the fieldwork who could help us identify the most suitable informant and location for our interviews. We also refurbished our stock of PPE and disinfecting equipment.

Four members of the AISdt project were directly involved in data collection in the field. We decided to form two research groups, so that we could work on-site in teams of two. Working in small groups made it easier to find a location where social distancing could be kept and lowered the probability of spreading the virus. For the same reason, we did not allow other people (family members or interested persons) to attend the recording sessions. We all did our utmost to keep transmission risks low. At the time of the fieldwork, all members of the team were fully vaccinated. As an additional precaution, each of us got tested regularly prior to and at the beginning and end of each interview cycle with a single informant. We also preferred to work with informants who were fully vaccinated themselves.

At the interview site, the area of direct interaction with the speaker (i.e., table, chairs, switches, door handles, etc.) was regularly sanitized with disinfectant wipes. The same was done with all the equipment. This included two notebook computers, a wireless mouse, a Zoom U-22 audio interface, a VT700H head-worn microphone, and a Panasonic HDC-SD90K camera with tripod. A 500 ml dispenser of sanitizing gel was kept on the table for all people to regularly clean their hands. As a rule, we sanitized our hands after every contact with an object present at the interview scene.

We defined the position of the three people taking part in the interview, keeping at least one metre apart, in accordance with the Italian measures on social distancing. The administration of the questionnaire via SpeechRecorder requires that the informant is placed in front of the computer screen, to see the image or text prompt, while the researcher starts and stops the recording of each answer. Both the informant and the linguist, therefore, must be able to see the screen. This was the most critical aspect of our collection setting. The person in charge of administering the questionnaire sat a metre away but slightly behind the informant, so that they could run the software with a mouse without ever getting into the informant’s safety range. Rather than relying on guesswork, we used a tape measure at the beginning of each session to make sure that the requirements on social distancing were met.10In some cases, we kept the tape measure on the table to remain aware of social distancing. The described arrangement always allowed us to work effortlessly and safely. However, we also use laptops that allow us to mirror the screen on other devices in the event that we do not have a direct line of sight to the informant screen. Research teams using machines that do not support this technology might consider adding an external monitor and an HDMI cable to the list of appliances to take to the field. The second researcher sat in front of the informant, on the opposite side of the table, at least one metre away. Their role was to compare in real time the responses elicited by the informant with the ones recorded in the original AIS, and to assess the informant’s active or passive competence of alternative lexemes.

Before starting the recording, a copy of the informed consent form (see Section 3.3) was given to the informant, while the researchers went through the content of each paragraph. By signing the informed consent form – with a disinfected pen for their exclusive use – they granted authorization to use the collected audio and video data for research purposes and acknowledged that the research team was taking all necessary measures to prevent the spread of the virus, but that they were aware of possible sanitary risks associated with social interactions amid a pandemic. Nonetheless, we made clear that they were free to leave the interview at any time if they believed that any of the stated conditions, especially those related to COVID-19 precautions, were not respected.

**Figure 3:**
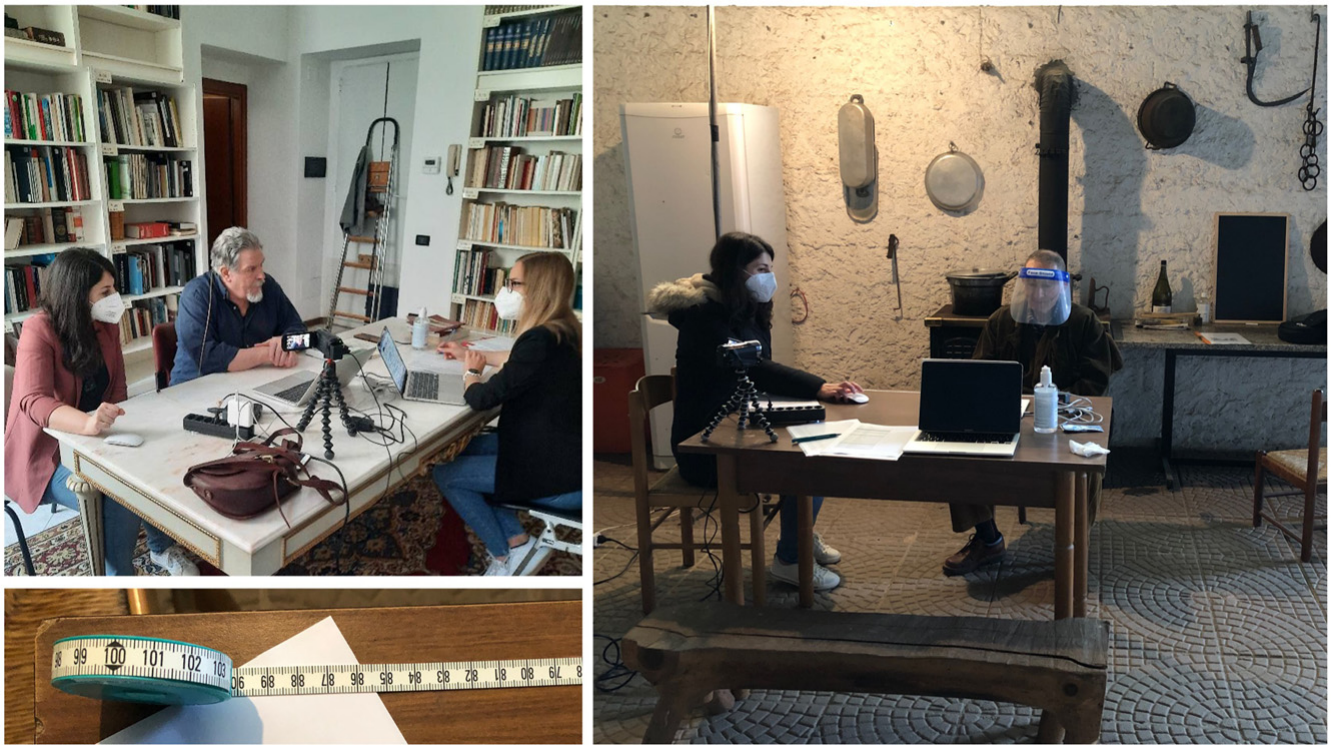
Social distancing and PPE equipment worn during the interviews for the AISdt project. *Left*, a photo from the fieldwork in Como (AIS data point 242); *right*, the recording in Vigevano (AIS data point 271); *below left*, a close-up of the tape measure used to verify that social distance requirements were met.

For our interviews, the informant wore a head-worn microphone. This is a very sensitive microphone, not compatible with the use of a face mask. However, its use is necessary to guarantee the audio recording quality required for the purposes of the AISdt project. As an alternative protective measure, we offered the informants the possibility of wearing a face shield, while the members of the collection team wore a FPP2 mask (equivalent to other international standards known as N95, KN95, and P2 masks), which we replaced at four-hour intervals (see Figure 3).11Although some informants opted to wear the face shield, all of them decided to remove it shortly after the beginning of the fieldwork session because they found it particularly uncomfortable. Our assessments of filtering face-pieces were based on the current state of knowledge and on the WHO COVID-19 advice for the public (WHO 2021a). The selected microphone was protected by a removable hard plastic cap which allows deep disinfection with disinfectant sprays, while the main body of the microphone could be easily cleaned with disinfectant wipes. This was done at the beginning and end of each recording session, and during breaks.12Studies suggest that the risk of contagion through contaminated surfaces is relatively low (Bueckert et al. 2020; CDC 2021) and dependent on the virus variant. However, as a protocol for AISdt data collection we decided to observe a one-week quarantine for the microphone before reusing it with a different informant. In cases where, for logistical reasons, we could not comply with this rule, we carried and used a second microphone.

As mentioned in Section 2, our interviews are also video-recorded. This was initially intended as a means of keeping record of all content elicited during the interview that does not specifically pertain to the questionnaire. When doing fieldwork amid a pandemic, it also became an additional protection instrument for the research team and the informant, as it recorded the behaviour of both parties throughout the interview and could be used as evidence of proper compliance with the rules.

## 5 Final remarks

From the beginning of September 2021 – when fieldwork activities were resumed – to the end of November 2021, the research team of the AISdt project carried out seven fieldwork campaigns. The data points investigated were Sant’ Omobono Terme (244), Branzi (236), Grosio (218), Bergamo (246), Vigevano (271), Cozzo (270), and Martinengo (254).13The numbers in parentheses refer to the data point identification number in the atlas. For their geographical distribution, please refer to Figure 2. The data collected on this occasion has already been the subject of studies and papers. These include Salvi (2023a), Salvi (2023b), Gasner and Salvi (2023), Gasner et al. (2023), Gasner (forthcoming), and Salvi (forthcoming). For an up-to-date list of the publications produced within the AISdt project, please refer to https://www.rose.uzh.ch/de/forschung/projekte/AIS,-the-digital-turn.html. In the context of these interviews alone, 12,160 responses were gathered, and these were transcribed and uploaded to the online database *AIS, reloaded* (Loporcaro et al. 2024); an average of 10 hours of recording were collected for each data point (75.48 hours in total).14All questionnaire data are freely available and downloadable from the project website, including the audio files. The data of the video recordings, on the other hand, are deposited at the Phonogram Archives of the University of Zurich and are available for consultation upon request. This occurred at a stage when, understandably, the discussion of the effects of the pandemic on linguistic research was still centred on remote data collection. Given the questionnaire and the informants targeted by the AISdt project, resuming face-to-face interviews at a time when the spread of the new SARS-CoV-2 was still very critical was a necessity rather than a choice. All the technical and methodological insights provided in the paper are the outcome of the research work carried out within our group, with the advice of medical, legal, and sound experts.

Did working under this new modality affect the quality of the data collected for the project? No. The protocols put into place to work safely during the pandemic had no direct effect on our research method and empirical approach to data elicitation. The correct use of PPE equipment and the sanitization of instruments and environments by the researchers allowed the informants to be safely recorded without them wearing a mask, which would on the contrary have interfered with the quality of our recordings.

Did the protocol interfere with the type of data collected? Partially. The decision to exclude other local people from the scene of the interview prevented the video-recording of any (semi-)spontaneous interaction between native speakers. Although this had no direct effect on the dataset for the AISdt database, this data would have been supplementary material that could have had good potential for ancillary studies.

Clearly, the positive conclusions drawn from the experience of the AISdt team are contingent on the aim of the investigation. However, we believe that the same setting and safety protocols can be successfully replicated – mutatis mutandis – in other studies.

Abstracting from the discussion of the results, there are other aspects that researchers should consider before returning to the field during a pandemic. Fieldwork under these exceptional conditions is exceptionally demanding. In addition to the many tasks required by data collection, the linguist now also has the responsibility of ensuring that all safety measures are constantly observed. On a formal level, by virtue of the agreement signed at the outset (Section 3.3), this responsibility was shared between interviewees and interviewers. However, in our experience, to think that the informant is as concerned as the research team about respecting the measures is a crude mistake. Out of the seven informants we worked with, only in two cases did the informant verify that we were vaccinated or consider wearing a face shield. Moreover, it often happened that, while focusing on the answers, speakers made unconscious gestures, such as touching their face or bringing their fingers to their mouth. This is totally understandable, as the informant is under pressure and focused on a different task during the interview, but it puts the researcher in a constant state of alertness. A further aspect that should not be underestimated is the physical fatigue caused by wearing an FFP2 mask for long time. For this reason, we found it useful to take 10-min breaks every hour to leave the premises of the interview, as we never removed the mask in the presence of the informant, not even to have a drink of water.

Resuming in-person collection activities was not a decision taken lightly, but we now believe that it was important for us as well as for the speakers, despite all the difficulties. In Lombardy, 89.63 % of deaths linked to COVID-19 occurred in the over-60 population (Regione Lombardia 2021). In many small communities, the documentation of the local dialect has taken on a whole new meaning for the informants, who felt invested in ensuring that signs of their generation’s culture and language remained.

We are aware that many of the measures we adopted to get back to fieldwork – such as the one-week quarantine for microphones – went beyond the requirements of authorities. However, the protocol described allowed us to work conscious of having made all possible efforts in terms of limiting the spread of the COVID-19 virus. Being so rigorous and deciding, for example, to continue to get tested before participating in the interview even when it was no longer required, actually made a difference in one case, preventing a COVID-positive member of the crew from putting a colleague and informant at risk. We are equally aware that many of the guidelines given in the article may seem obvious or outdated in an era in which we have come to know and live with this virus. Most of all, this article is a testimony to how linguistic research in the field had to adapt to unprecedented conditions and in a very vulnerable linguistic landscape. Hopefully, it will be of help to linguists to plan future in-person data collection campaigns, and it will restore confidence in the possibility of returning safely to the field amid pandemic events.

## References

[j_lingvan-2021-0155_ref_001] Bueckert Max, Gupta Rishi, Gupta Aditi, Garg Mohit, Mazumder Asit (2020). Infectivity of SARS-CoV-2 and other coronaviruses on dry surfaces: Potential for indirect transmission. *Materials*.

[j_lingvan-2021-0155_ref_002] CDC (2021). Science brief: SARS-CoV-2 and surface (fomite) transmission for indoor community environments. Centers for Disease Control and Prevention. ..

[j_lingvan-2021-0155_ref_003] Draxler Christoph, Jänsch Klaus, Lino Maria Teresa, Xavier Maria Francisca, Ferreira Fátima, Costa Rute, Silva Raquel (2004). SpeechRecorder – a universal platform independent multi-channel audio recording software. *Proceedings of the Fourth International Conference on Language Resources and Evaluation (LREC’04)*.

[j_lingvan-2021-0155_ref_004] Gasner Lisa (Forthcoming). *Aspetti di morfologia verbale dei dialetti lombardi*.

[j_lingvan-2021-0155_ref_005] Gasner Lisa, Salvi Adriano (2023). AIS, the digital turn: Atlanti dialettali a cento anni di distanza e il ruolo delle nuove tecnologie. ..

[j_lingvan-2021-0155_ref_006] Gasner Lisa, Donzelli Giulia, Idone Alice, Salvi Adriano (2023). Il dialetto di Vigevano nei dati AIS e AIS, *the digital turn*: Uno studio sul mutamento del vigevanese negli ultimi cento anni. *Fluidità ed evanescenze dialettali in Lombardia nel XX e XXI secolo: Il dialetto vigevanese e l’areale del Lombardo Occidentale. Proceedings of “Convegno Internazionale di Dialettologia della Società Storica Vigevanese”*.

[j_lingvan-2021-0155_ref_032] ISTAT (2020). Resident population in Lombardy on 1st January 2020. Database of the Italian National Institute of Statistics.

[j_lingvan-2021-0155_ref_007] Jaberg Karl, Jud Jakob (1928–1940). *Sprach- und Sachatlas Italiens und der Südschweiz/Atlante linguistico ed etnografico dell’Italia e della Svizzera meridionale (AIS)*.

[j_lingvan-2021-0155_ref_008] Loporcaro Michele (2013). *Profilo linguistico dei dialetti italiani*.

[j_lingvan-2021-0155_ref_009] Loporcaro Michele, Stephan Schmid, Diego Pescarini, Graziano Tisato, Donzelli Giulia, Stefano Negrinelli, Zanini Chiara (2019). AIS, reloaded (AISr). University of Zurich. https://www.rose.uzh.ch/de/forschung/projekte/AIS-reloaded.html.

[j_lingvan-2021-0155_ref_010] Loporcaro Michele, Donzelli Giulia, Gasner Lisa, Idone Alice, Salvi Adriano, Tisato Graziano (2021a). AIS, the digital turn (AISdt). University of Zurich. ..

[j_lingvan-2021-0155_ref_011] Loporcaro Michele, Schmid Stephan, Zanini Chiara, Pescarini Diego, Donzelli Giulia, Negrinelli Stefano, Tisato Graziano, Thibault André, Avanzi Matthieu, Vecchio Nicolas Lo, Millour Alice (2021b). AIS, reloaded: A digital dialect atlas of Italy and southern Switzerland. *Nouveaux regards sur la variation dialectale*.

[j_lingvan-2021-0155_ref_033] Loporcaro Michele, Stephan Schmid, Diego Pescarini, Graziano Tisato, Giulia Donzelli, Lisa Gasner, Alice Idone, Stefano Negrinelli, Adriano Salvi, Chiara Zanini (2024). *AIS, reloaded (AISr)*.

[j_lingvan-2021-0155_ref_012] Maiden Martin, Parry Mair (1997). *The dialects of Italy*.

[j_lingvan-2021-0155_ref_013] Ministero della Salute (2021). Report vaccini anti COVID-19. ..

[j_lingvan-2021-0155_ref_014] Openpolis (2021). Coronavirus, l’elenco completo degli atti. ..

[j_lingvan-2021-0155_ref_015] Pellegrini Giovan Battista (1977). *Carta dei dialetti d’Italia*.

[j_lingvan-2021-0155_ref_016] Presidenza del Consiglio dei Ministri (2021). Coronavirus, le misure adottate dal Governo Italiano. ..

[j_lingvan-2021-0155_ref_017] Protezione Civile (2021a). Coronavirus: La situazione. Dipartimento della Protezione Civile, Governo Italiano. ..

[j_lingvan-2021-0155_ref_018] Protezione Civile (2021b). Coronavirus: Mappe. Dipartimento della Protezione Civile, Governo Italiano. ..

[j_lingvan-2021-0155_ref_019] Protezione Civile (2021c). Dati COVID-19 Italia. Dipartimento della Protezione Civile, Governo Italiano. ..

[j_lingvan-2021-0155_ref_020] Regione Lombardia (2021). Dashboard Covid-19. Updated 29 November 2021. ..

[j_lingvan-2021-0155_ref_021] Salvi Adriano, Faraoni Vincenzo, Filipponio Lorenzo, Paciaroni Tania, Schmid Stephan (2023a). Analisi sperimentale della qualità vocalica nel dialetto di Berbenno (BG). *Prospettive di ricerca linguistica italiana e romanza: Studi offerti a Michele Loporcaro dagli allievi e dai collaboratori zurighesi*.

[j_lingvan-2021-0155_ref_022] Salvi Adriano (2023b). Sull’articolo determinativo femminile plurale e sulla coniugazione interrogativa nel dialetto di Berbenno (BG). ..

[j_lingvan-2021-0155_ref_023] Salvi Adriano (Forthcoming). *Il dialetto di Berbenno (BG): Fonologia, morfologia, sintassi di un dialetto bergamasco*.

[j_lingvan-2021-0155_ref_024] Sennheiser (2021). How to maintain good microphone hygiene, version 1.6 (April 2021). ..

[j_lingvan-2021-0155_ref_025] Statista (2021). Coronavirus (COVID-19) cases in Italy as of November 28, 2021, by region. ..

[j_lingvan-2021-0155_ref_026] WHO (2021a). Advice for the public: Coronavirus disease (COVID-19). World Health Organization. ..

[j_lingvan-2021-0155_ref_027] WHO (2021b). COVID-19 measures. World Health Organization. ..

[j_lingvan-2021-0155_ref_028] Wikipedia (2021). COVID-19 pandemic in Italy. ..

[j_lingvan-2021-0155_ref_029] Wilkinson Mark D., Dumontier Michel, Jan Aalbersberg I. Jsbrand, Appleton Gabrielle, Axton Myles, Baak Arie, Blomberg Niklas, Boiten Jan-Willem, da Silva Santos Luiz Bonino, Bourne Philip E., Bouwman Jildau, Brookes Anthony J., Clark Tim, Crosas Mercè, Dillo Ingrid, Dumon Olivier, Edmunds Scott, Evelo Chris T., Finkers Richard, Gonzalez-Beltran Alejandra, Gray Alasdair J. G., Groth Paul, Goble Carole, Grethe Jeffrey S., Heringa Jaap, ’t Hoen Peter A. C., Hooft Rob, Kuhn Tobias, Kok Ruben, Kok Joost, Lusher Scott J., Martone Maryann E., Mons Albert, Packer Abel L., Persson Bengt, Rocca-Serra Philippe, Roos Marco, van Schaik Rene, Sansone Susanna-Assunta, Schultes Erik, Sengstag Thierry, Slater Ted, Strawn George, Swertz Morris A., Thompson Mark, van der Lei Johan, van Mulligen Erik, Velterop Jan, Waagmeester Andra, Wittenburg Peter, Wolstencroft Katherine, Zhao Jun, Mons Barend (2016). The FAIR Guiding Principles for scientific data management and stewardship. *Scientific Data*.

[j_lingvan-2021-0155_ref_030] Winfield Nicole (2022). Italy targets the unvaccinated with new virus restrictions. *Associated Press*.

[j_lingvan-2021-0155_ref_031] Worldometer (2021). Reported cases and deaths by country or territory. ..

